# Highly Efficient Removal of Nitrate and Phosphate to Control Eutrophication by the Dielectrophoresis-Assisted Adsorption Method

**DOI:** 10.3390/ijerph19031890

**Published:** 2022-02-08

**Authors:** Jiaxi Li, Qinghao Jin, Yuran Liang, Junfeng Geng, Jianxin Xia, Huiying Chen, Miaoying Yun

**Affiliations:** 1 College of Life and Environmental Science, Minzu University of China, Beijing 100081, China; 21302390@muc.edu.cn (J.L.); kingsman@muc.edu.cn (Q.J.); 19301170@muc.edu.cn (Y.L.); 2 Institute for Materials Research and Innovation, Institute for Renewable Energy and Environmental Technologies, University of Bolton, Bolton BL3 5AB, UK; J.Geng@bolton.ac.uk

**Keywords:** dielectrophoresis, adsorption, nitrate, phosphate, plant ash, eutrophication control

## Abstract

The removal of excessive amounts of nitrate and phosphate from water sources, especially agricultural wastewater, has been of high significance to control eutrophication in aquatic systems. Here, a new method is reported for the removal of nitrate and phosphate simultaneously from wastewater based on the combination of the solution-phased adsorption (ADS) and dielectrophoresis (DEP) techniques. The plant ash was first selected as the adsorbent by screening tests, followed by a systematic investigation of using the adsorbent to remove nitrate and phosphate from wastewater under various experimental conditions, including the testing of adsorbent dosage, pretreatment time, water flow rate, and electrode voltage. The analysis of the adsorbent particles was also performed by scanning electron microscope (SEM) analysis, the energy dispersive X-ray spectroscopy (EDX) test, and the measurement of Zeta potentials. Compared with the ADS method alone, the introduction of DEP into the purification process has greatly increased the removal rate by 66.06% for nitrate and 43.04% for phosphate, respectively. In the meantime, it is observed that the processing time has been greatly reduced by 92% with the assistance of DEP.

## 1. Introduction

As is well known, nitrogen (N) and phosphorus (P) are the primary nutrients in lakes, rivers, and wetlands. Nitrogen and phosphorus support the growth of algae, bacteria, and aquatic plants in water, but too much of these elements can be harmful. When a water body receives an excessive amount of N or P, it can be polluted by the overgrowth of algae and other aquatic plants. This eutrophication process can consequently reduce the dissolved oxygen content, suffocate fish and other aquatic life, minimize the water transparency, reduce the overall water quality, and damage the ecological balance. Some algae can even produce toxins that are harmful to livestock and human health [1].

In water, nitrogen and phosphorus often exist in the form of nitrates and phosphates—the organic or inorganic compounds that contain nitrogen or phosphorus. Nitrate in water is primarily used by algae and fish to synthesize protein, but excessive nitrate in water can also cause severe illness in infants and domestic animals. Phosphate is a vital nutrient for converting sunlight into usable energy, which is essential for cellular growth and reproduction [2,3,4,5,6,7,8]. Common sources of excess nitrates and phosphates reaching lakes and streams include septic systems, agricultural fertilizers, animal food and manure, domestic sewage, industrial waste waters, sanitary landfills, and garbage dumps [9,10,11,12]. The question is how to remove excessive nitrates and phosphates from wastewater, rivers, or lakes in order to protect the environment?

Current methods for removing nitrates and phosphates from water are mainly the following ones: the adsorption method [13], biological treatment [14], active metal reduction [15], electrochemical catalytic reduction [16], and chemical precipitation [17,18]. Although all of these approaches are useful, each of them has its drawbacks. For example, the chemical precipitation method can easily cause secondary pollution [19], while the biological treatment technique requires a long reaction period with stringent conditions [20]. In comparison, adsorption is a relatively convenient and reliable technique, but it normally has a long treatment cycle and low efficiency, and if one wants to use an efficient adsorbent for the adsorption, the cost is usually high [21].

Here we report the dielectrophoresis-assisted adsorption (ADS/DEP) method to effectively remove nitrate and phosphate from wastewater. The DEP technique has been used in a number of fields such as biology, medicine, and materials [22], as well as in the field of water pollution control. We have previously conducted a number of studies by using the ADS/DEP method for the removal of cations, such as cadmium [23] and ammonia nitrogen [24], and anion of arsenic [25], and coexisting cations of cadmium and lead at the same time [26] from wastewater. Based on the above research results, the present report is the first to use the ADS/DEP technique to remove coexisting anions, nitrate, and phosphate, which are the main pollutants of eutrophication.

## 2. Materials and Methods

### 2.1. Materials

A nitrate solution was prepared by dissolving an appropriate amount of potassium nitrate (KNO_3_, analytical reagent, Sinopharm Chemical Reagent Co., Ltd., Beijing, China) in deionized water (about 1 μS/cm). A phosphate solution (pH = 6.89, where the phosphate is in the main form of H_2_PO_4_^−^ [27]) was prepared by dissolving an appropriate amount of potassium dihydrogen phosphate (KH_2_PO_4_, analytical reagent, Sinopharm Chemical Reagent Co., Ltd., Beijing, China) in ultrapure water.

### 2.2. Adsorption Experiments

Plant ash (Shandong, China), activated carbon (Sinopharm Chemical Reagent Co., Ltd., Beijing, China), graphite powder (Shanghai Jingrui Scientific Instrument Co., Ltd., Shanghai, China), and rice husk charcoal (Hebei, China) were screened as adsorbents. The simulated solutions with a predetermined amount of adsorbent were added to a conical flask, and the mixture was thermostatically stirred. After a period of adsorption, the suspension was filtered. The concentration of nitrate and phosphate solutions was determined by ultraviolet spectrophotometry and the phosphomolybdenum blue spectrophotometric method with an ultraviolet-visible spectrophotometer (JV-650, Japan). The removal efficiency was calculated as

(1) $$Re\left(\%\right)=\frac{C_{0}-C_{i}}{C_{0}}\times 100$$

where Re is the removal and $C_{0}$ and $C_{i}$ represent the initial and sample concentration, respectively.

### 2.3. Dielectrophoresis Experiments

**Figure 1:**
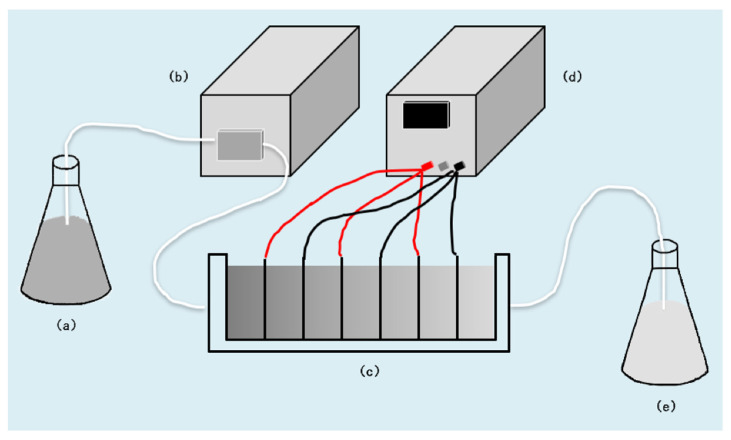
A schematic diagram showing the device layout used in the experiments: (**a**) suspension, (**b**) pump, (**c**) capture pool, (**d**) power, and (**e**) effluent.

DEP experiments were conducted with a house-developed device as shown in Figure 1. The simulated solution (30 mg/L nitrate solution [28] and 100 mg/L phosphate solution [29]) after adsorption pretreatment was first filtered through the electrode wire mesh and then pumped into the dielectric pool through a peristaltic pump. A power supply provided a voltage through the wires to the stainless steel wire mesh electrodes in the dielectric pool. The electrodes, the size of which was 35 × 50 mm with 0.18 mm mesh size, were installed in the capture pool through slots and alternately connected to the positive and negative poles of the power supply. The solution treated by DEP was forced into the collection tank under the peristaltic pump. The calculation of removal efficiency was the same as that of the adsorption experiment. The device can be used for secondary treatment to further improve the ion removal rate [25].

Dielectrophoresis is the motion of particles caused by the polarization of non-uniform electric fields. Pohl established the traditional DEP force model [30]. For a spherical particle, the dielectric power that it receives in the non-uniform electric field can be expressed as

(2) $$\;F_{DEP}=2\pi R^{3}\varepsilon_{m}Re\left[K\left(\omega \right)\right]\nabla E^{2}$$

where *R* denotes the radius of the particle, *ε_m_* the real part of the medium permittivity, and ∇*E*^2^ the squared electric field intensity, while the real part of the Clausius–Mossotti factor, *Re*[*K*(*ω*)], is defined as

(3) $$\;K\left(\omega \right)=\frac{\varepsilon_{p}^{*}-\varepsilon_{m}^{*}}{\varepsilon_{p}^{*}+2\varepsilon_{m}^{*}}$$

where ε_p_* and ε_m_* are the complex permittivity of the particles and the liquid, respectively. When [*K*(*ω*)] > 0, the polarizability of the particles would be greater than that of suspension media. The direction of the DEP force is, in this case, along the direction of the electric field gradient, and the particles move towards the region with the strongest electric field, which is a positive dielectrophoresis (pDEP). When [*K*(*ω*)] < 0, on the contrary, the particles will move towards the region with weak electric field strength, and this phenomenon is a negative dielectrophoresis (nDEP).

### 2.4. Characterization of Adsorbents

Analysis of the adsorbent samples was conducted by scanning electron microscope (SEM) and the energy dispersive X-ray spectroscopy (EDX) (Quanta 650FEG, FEI, Hillsboro, OR, USA). The electrical properties of the adsorbent surface before and after the treatment were compared by measuring the Zeta potential (Nano-ZS; Malvern, UK).

## 3. Results and Discussion

### 3.1. Screening Test of Adsorbents

**Figure 2:**
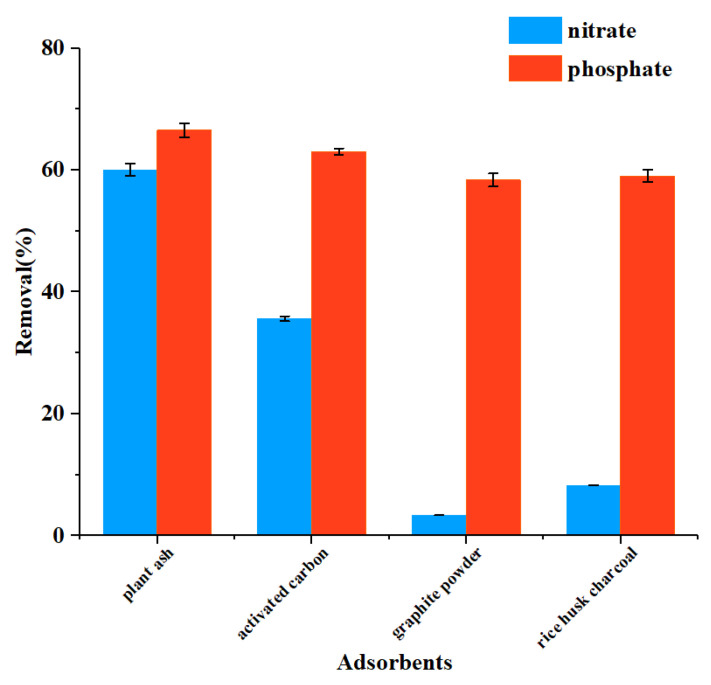
Effects of different adsorbents at 100 g/L on the removal of nitrate and phosphate (100 mL, 100 mg/L) after 24 h adsorption.

The adsorbent screening result is shown in Figure 2. Under identical experimental conditions, it was observed that each adsorbent showed a better adsorption effect on phosphate than on nitrate. Among the selected adsorbents, plant ash showed the best adsorption effect: 60.04% for nitrate and 66.57% for phosphate. Plant ash is the ash produced by burning plants, and its main component is potassium carbonate. Plant ash is an inorganic farmyard manure with a wide range of sources, a low cost, and obvious fertilizer efficiency [13,31]. So, it is expected to be made into compound fertilizers for reuse after adsorbing nitrate and phosphate. Studies have shown that plant ash is a good adsorbent, and its particle size is mainly in the range of 10 to 100 μm, and there may be functional groups such as carboxyl and hydroxyl groups [32,33]. Adsorption of nitrate and phosphate on plant ash followed pseudo-second-order adsorption kinetics, meaning that the adsorption rate is mainly affected by the rate of chemical bond formation. It was found that nitrate adsorption on plant ash was monolayer adsorption. For phosphate, the adsorption process involves both monolayer and multimolecular layer adsorption (Tables S1 and S2). In the end, we selected plant ash as the adsorbent because it is low cost and easy to obtain, and has a high adsorption efficiency.

### 3.2. Optimization of the Adsorbent Pretreatment Time

**Figure 3:**
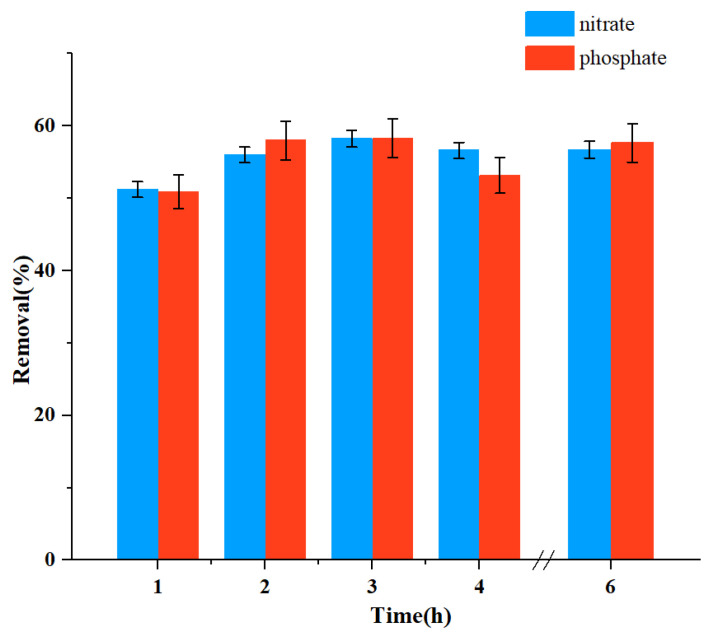
Effect of time on the removal efficiency of nitrate and phosphate.

To find out the best length of treatment time using the adsorbent, we added 10 g/L of the plant ash to the simulated solution and stirred it for 1 h, 2 h, 3 h, 4 h, and 6 h, respectively, before pumping the solution into the DEP apparatus. The voltage was controlled at 15 V [24], and the flow rate was 0.503 L/h. As shown in Figure 3, the removal rates increased slightly with the increase in time from 1 h to 6 h (the efficiency for nitrate increased from 51.18% to 58.15%, and for phosphate it increased from 50.89% to 58.34%), which shows that the time factor played a role there, but it was not very significant after the initial 1 h of adsorption. Therefore, in the subsequent experiment, we used 1 h as the adsorption pretreatment time.

### 3.3. Optimization of Adsorbent Dosage

**Figure 4:**
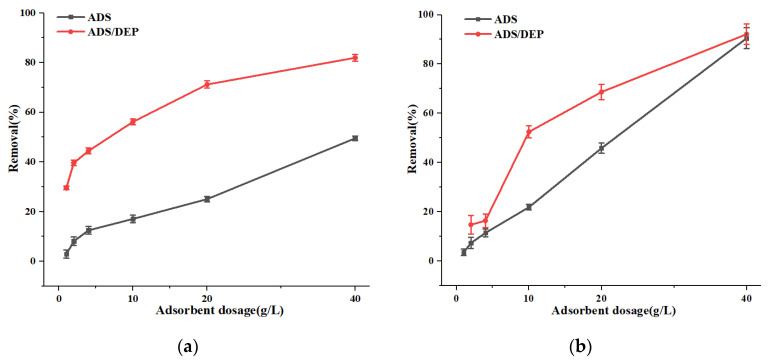
The effect of absorbent dosage on removal efficiency when using adsorption (ADS) alone (pH = 7, ADS time = 24 h, volume = 50 mL) or dielectrophoresis-assisted adsorption (ADS/DEP) (ADS time = 1 h, volume = 500 mL, voltage = 15 V, flow = 0.503 L/h): (**a**) nitrate and (**b**) phosphate.

Plant ash at concentrations of 1 g/L, 2 g/L, 4 g/L, 10 g/L, 20 g/L, and 40 g/L was added to the simulated solution to test the dosage effect on the removal efficiency under 1 h adsorption treatment time. The results are shown in Figure 4. The removal efficiency clearly increased with the increase in adsorbent dosage. While considering this result and the cost factor, we decided to use a dosage of 10 g/L of adsorbent in subsequent experiments.

### 3.4. Effect of the DEP Process

As shown in Figure 4, after introducing DEP into the system, the adsorption efficiency increased by 39.22% for nitrate and 30.40% for phosphate, respectively, at the 10 g/L adsorbent level, compared with the use of adsorption alone. Moreover, the treatment time reduced by 92% (the adsorption time for ADS alone was 24 h, but it was less than 2 h for ADS/DEP). This remarkable enhancement may be explained as follows: the non-uniform electric field generated in the DEP device polarizes the adsorbent and induces a dipole moment on each adsorbent particle. Thus, the electric field exerts an unbalanced force on the particles, driving them along the electric field gradient in the solution. Particles near the electrode were first captured by the electrode. Due to the strong electric field in the cross-wire region of the mesh electrode and a possible polarization induction effect between adjacent adsorbent particles, other particles near the junction could be polarized and captured by the electrode [24]. In this way, more and more adsorbent particles were effectively trapped by the electrodes over time, which greatly helped increase the removal efficiency and reduce the treatment time.

### 3.5. Effect of the Flow Rate

**Figure 5:**
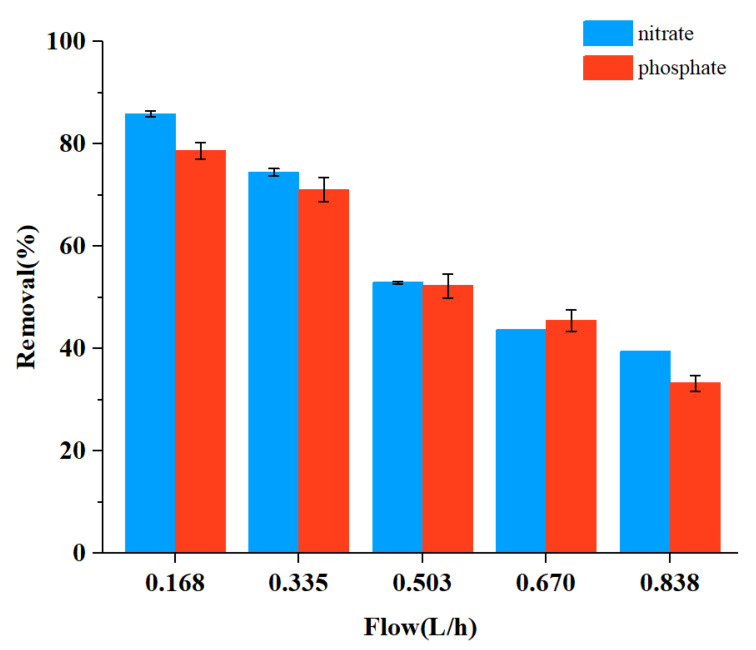
Effect of flow rate on the removal efficiency of nitrate and phosphate.

To test the flow rate effect, 10 g of plant ash was added to 1 L of simulated solution, the absorption treatment time was set at 1 h, and the voltage was 15 V. The flow rate was controlled at 0.168 L/h, 0.335 L/h, 0.503 L/h, 0.670 L/h, and 0.838 L/h. The results are shown in Figure 5. It can be seen that with the increase of flow rate, the removal efficiency decreased. The mechanism of this flow rate effect may be understood from the nature of the DEP force. Because the DEP force was a short-distance force, a smaller flow rate would mean a longer processing time, during which more plant ash particles could have a chance to be moved closer to the electrodes, be trapped on the electrodes, and consequently be removed from the solution [24].

### 3.6. Optimization of the Voltage

**Figure 6:**
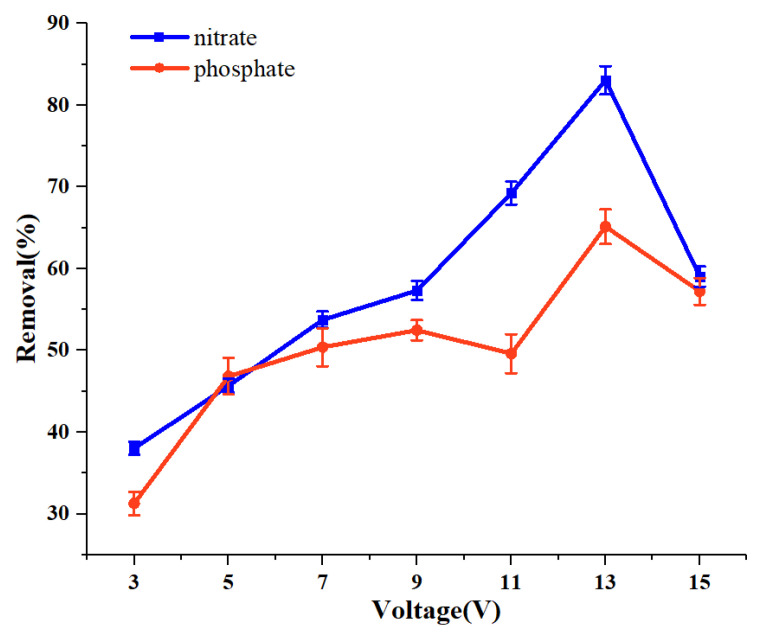
Effect of voltage on the removal efficiency of nitrate and phosphate.

To test the voltage effect, the voltage was set at 3 V, 5 V, 7 V, 9 V, 11 V, 13 V, and 15 V, respectively, while the adsorbent dosage and treatment time were kept at the same levels as described in Section 3.5, except for the flow rate which was set at 0.503 L/h. The result is shown in Figure 6. It can be seen that when the voltage increased, the removal efficiency increased initially but decreased later. The removal rate reached the highest at 13 V for both nitrate and phosphate, which is clearly the optimal capture voltage. This voltage effect can be explained by the DEP principle. Polarizable particles have their own characteristic voltage, at which the DEP force reaches the strongest and the particles can be most easily captured. Higher than this voltage, the electric field of electrode is too strong, and the particles will leave the surface of the wire mesh electrode and be taken away by the fluid medium. In addition, the electrode electrolysis will be very intense under high voltage, which affects the capture of plant ash particles [24,34]. Interestingly, the DEP removal of nitrate and phosphate has shown the same optimal capture voltage, which provides a good basis for the simultaneous removal of nitrate and phosphate from wastewater.

### 3.7. Analysis of Plant Ash Particles by SEM and EDX

**Figure 7:**
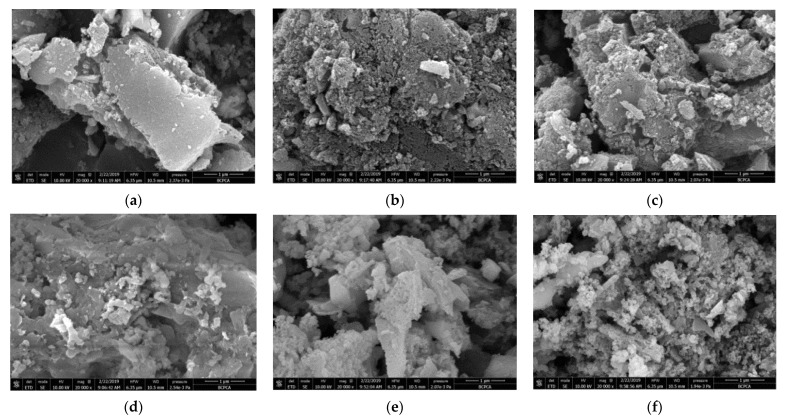
SEM images of the plant ash particles after different treatments: (**a**) before ADS of nitrate; (**b**) after ADS of nitrate; (**c**) after DEP of nitrate; (**d**) before ADS of phosphate; (**e**) after ADS of phosphate; and (**f**) after DEP of phosphate.

SEM analyses were performed for the particles before and after the adsorption treatment with ADS alone and the combination of ADS and DEP. The samples were collected after each experiment, and dried in a desiccator for 24 h. Figure 7 shows the morphologies of plant ash after different treatments. It was noted that compared with the original particles and the ADS-treated particles, the surface morphology of the DEP-treated particles changed greatly. After DEP treatment, the surface of the plant ash particles was contracted, which showed a compact arrangement. This may be because the original particles are broken into smaller particles under the action of the DEP force, so the specific surface area and adsorption sites increased, and consequently, the removal rate increased [26].

EDX was used to evaluate the adsorption of nitrate and phosphate on the plant ash. The results indicated that the weight percentage of the phosphorus absorbed on plant ash increased from 0.16% (by only ADS) to 0.55% (after ADS/DEP), which is positively correlated with the growth of removal rate. This confirms that the DEP process facilitated more phosphate to be bonded to the plant ash surface. Due to the weak detecting ability of the X-ray energy spectrum for light elements with atomic numbers below 11 (Na), nitrogen was not detected on the surface of the plant ash before and after DEP.

### 3.8. Characterization of Zeta Potential

**Figure 8:**
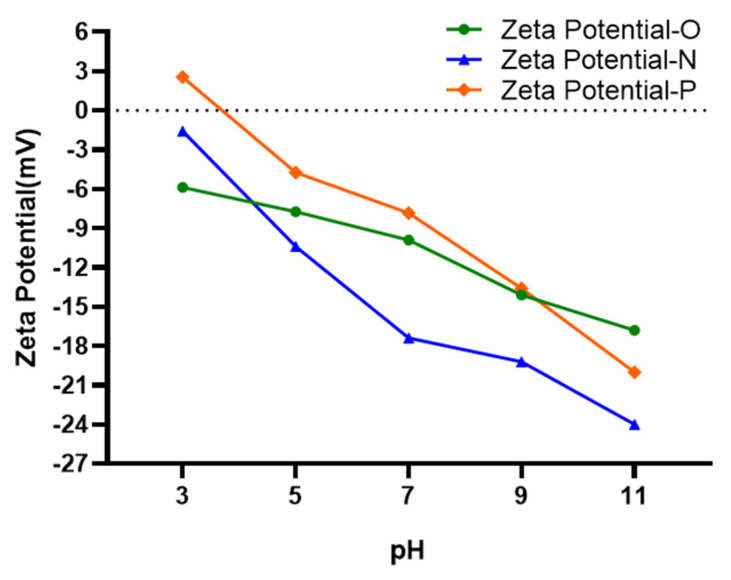
Zeta potentials of plant ash as a function of pH.

Zeta potential refers to the potential generated by the surface charge of particles dispersed in solution. The changes in zeta potential with the pH of the original plant ash sample and the sample treated by ADS/DEP are plotted in Figure 8. For the original plant ash particles (Zeta Potential-O), the zeta potential is negative based on the degree of ionization of OH^−^ functional groups on the adsorbent surface [35]. The zeta potential decreases with the increase of pH value. This is because the OH^−^ in the solution helps to increase the concentration of negative charge on the surface of the adsorbent [35].

After ADS/DEP, the zeta potentials of nitrate (Zeta Potential-N) and phosphate (Zeta Potential-P) changed significantly. Under acidic conditions, Zeta Potential-N and Zeta Potential-P both are higher than the Zeta Potential-O, probably because the positive cations in the solution, such as H^+^ and K^+^, were adsorbed by plant ash particles. The decrease in the potential under alkaline conditions may be due to the increase in negative charge such as OH^−^ on the particle’s surface. It is worth noting that under the current experimental conditions (with pH near the neutral range), Zeta Potential-N is lower than Zeta Potential-O, because nitrate is anions with negative charge, and its capture on the surface of the plant ash will cause the zeta potential to drop. However, Zeta Potential-P is a little higher than Zeta Potential-O, probably due to the positive cations absorbed by H_2_PO_4_^−^ and HPO_4_^2−^.

## 4. Conclusions

In this work, plant ash was selected as the best adsorbent material due to its better adsorption capacity, low cost, ease of handling, and reusability. Several parameters related to the removal efficiency were systematically optimized. The removal efficiency of nitrate and phosphate with ADS/DEP increased by 66.06% and 43.04%, respectively, compared to the ADS method alone. In addition, the processing time reduced by 92%, while the method parameters such as pretreatment time (1 h), adsorbent dose (10 g/L), flow rate (0.503 L/h), voltage (13 V), etc., were maintained. The results allowed us to conclude that the ADS/DEP method could provide an effective and viable means of removing nitrate and phosphate from wastewater in the future to control eutrophication on a large scale.

## Data Availability

Data is contained within the article.

## Supplementary Materials

The following supporting information can be downloaded at https://www.mdpi.com/article/10.3390/ijerph19031890/s1: Table S1: Values of kinetics parameters: k_1_ is the pseudo-first-order rate constant, k_2_ is the pseudo-second-order rate constant, q_e_ is the equilibrium adsorption capacity, R^2^ is the linear fit correlation coefficient; Table S2: Values of parameters related adsorption isotherms: Q_max_ and b are Langmuir constant for adsorption capacity and adsorption rate, K_f_ and n are Freundlich constant for adsorption capacity and adsorption intensity.
