# Supplementary material for: Highly Efficient Removal of Nitrate and Phosphate to Control Eutrophication by the Dielectrophoresis-Assisted Adsorption Method
Source: Int J Environ Res Public Health. 2022 Feb 8;19(3):1890. doi: 10.3390/ijerph19031890 (PMC8835578; doi:10.3390/ijerph19031890)
Supplement: Supplementary file 1 [file ijerph-19-01890-s001.zip › ijerph-1563155-supplementary.pdf]

Supplementary material

The preliminary exploration of the mechanism of plant ash adsorbing nitrate and phosphate was conducted. In adsorption kinetic study, 50 mL of nitrate (30 mg/L) and phosphate (100 mg/L) solution having 0.5 g plant ash were stirred for 5, 10, 20, 40, 60, 90, 120, 180, 240, 360, 480, 720 and 1440 min respectively at 25 °C, and the concentration was determined after filtration. Both kinetics models were used for the analysis of adsorption data of nitrate and phosphate [1]. The result was shown in Table S1. It revealed that the adsorption of nitrate and phosphate on plant ash followed pseudo-second-order adsorption kinetic, meaning that the adsorption rate is mainly affected by the rate of chemical bond formation.

Table S1. Values of kinetics parameters: k_1 is the pseudo-first-order rate constant, k_2 is the pseudo-second-order rate constant, q_e is the equilibrium adsorption capacity, R^2 is the linear fit correlation coefficient.

Adsorption kinetics models	Pseudo-first-order kinetic			Pseudo-second-order kinetic		
	k_1	q_e(mg/g)	R^2	k_2	q_e(mg/g)	R^2
nitrate	−0.00415	1.00	0.846	0.0100	1.72	0.994
phosphate	-0.000681	3.51	0.833	0.171	5.959	0.979

Nitrate and phosphate solutions (50 ml) having 0.5 g of plant ash with concentrations of 10, 20, 40, 60, 100, 150, 200 and 300 mg/L were stirred at 25 °C for 24 h, and the concentration was determined after filtration. Langmuir and Freundlich isotherm were applied to check the adsorption behavior [1]. The obtained values are given in Table S2. It was found that nitrate adsorption on plant ash followed the Langmuir isotherm model and the process was monolayer adsorption. For phosphate, however, the adsorption followed Langmuir and Freundlich isotherm models, while the process involves both monolayer and multimolecular layer adsorption.

Table S2. Values of parameters related adsorption isotherms: Q_{max} and b are Langmuir constant for adsorption capacity and adsorption rate, K_f and n are Freundlich constant for adsorption capacity and adsorption intensity.

Adsorption isotherm model	Langmuir isotherm			Freundlich isotherm		
	Q_{max}(mg/g)	b(L/mg)	R^2	K_f	n	R^2
nitrate	2.252	0.029	0.997	0.203	2.27	0.928
phosphate	4.329	0.008	0.993	0.071	1.437	0.982

1. C. Bhan, S. Jiwan, C. S. Yogesh. Development of adsorbent from Mentha plant ash and its application in fluoride adsorption from aqueous solution: a mechanism, isotherm, thermodynamic, and kinetics studies. Int. J. Phytoremediat.2021,1-11.
